# hERG, *Plasmodium* Life Cycle, and Cross Resistance Profiling of New Azabenzimidazole Analogues of Astemizole

**DOI:** 10.1021/acsmedchemlett.3c00496

**Published:** 2024-03-18

**Authors:** Dickson Mambwe, Dina Coertzen, Meta Leshabane, Mwila Mulubwa, Mathew Njoroge, Liezl Gibhard, Gareth Girling, Kathryn J. Wicht, Marcus C. S. Lee, Sergio Wittlin, Diogo Rodrigo Magalhães Moreira, Lyn-Marie Birkholtz, Kelly Chibale

**Affiliations:** † Department of Chemistry, University of Cape Town, Rondebosch, 7701 Cape Town, South Africa; Ω Department of Biochemistry, Genetics & Microbiology, Institute for Sustainable Malaria Control, University of Pretoria, Private Bag X20, Hatfield, 0028 Pretoria, South Africa; § Drug Discovery and Development Centre (H3D), DMPK & Pharmacology, University of Cape Town, Observatory, 7925 Cape Town, South Africa; Ψ Centro de Pesquisas Gonçalo Moniz, Fundação Oswaldo Cruz (Fiocruz), Instituto Gonçalo Moniz, CEP 40296-710 Salvador, Brazil; ∥ Swiss Tropical and Public Health Institute, Socinstrasse 57, 4002 Basel, Switzerland; ⊥ University of Basel, 4003 Basel, Switzerland; ⧫ Wellcome Sanger Institute, Wellcome Trust Genome Campus, Hinxton CB10 1SA, United Kingdom; Δ Biological Chemistry and Drug Discovery, School of Life Sciences, University of Dundee, Dundee DD1 4HN, Scotland, United Kingdom; Π South African Medical Research Council Drug Discovery and Development Research Unit, University of Cape Town, Rondebosch, 7701 Cape Town, South Africa; ‡ Institute of Infectious Disease and Molecular Medicine, University of Cape Town, Rondebosch, 7701 Cape Town, South Africa

**Keywords:** Astemizole, *Plasmodium falciparum*, *Plasmodium berghei*, repositioning, human ether-á-go-go-related gene (hERG), gametocytocidal, liver-stage activity, resistance phenotypes

## Abstract

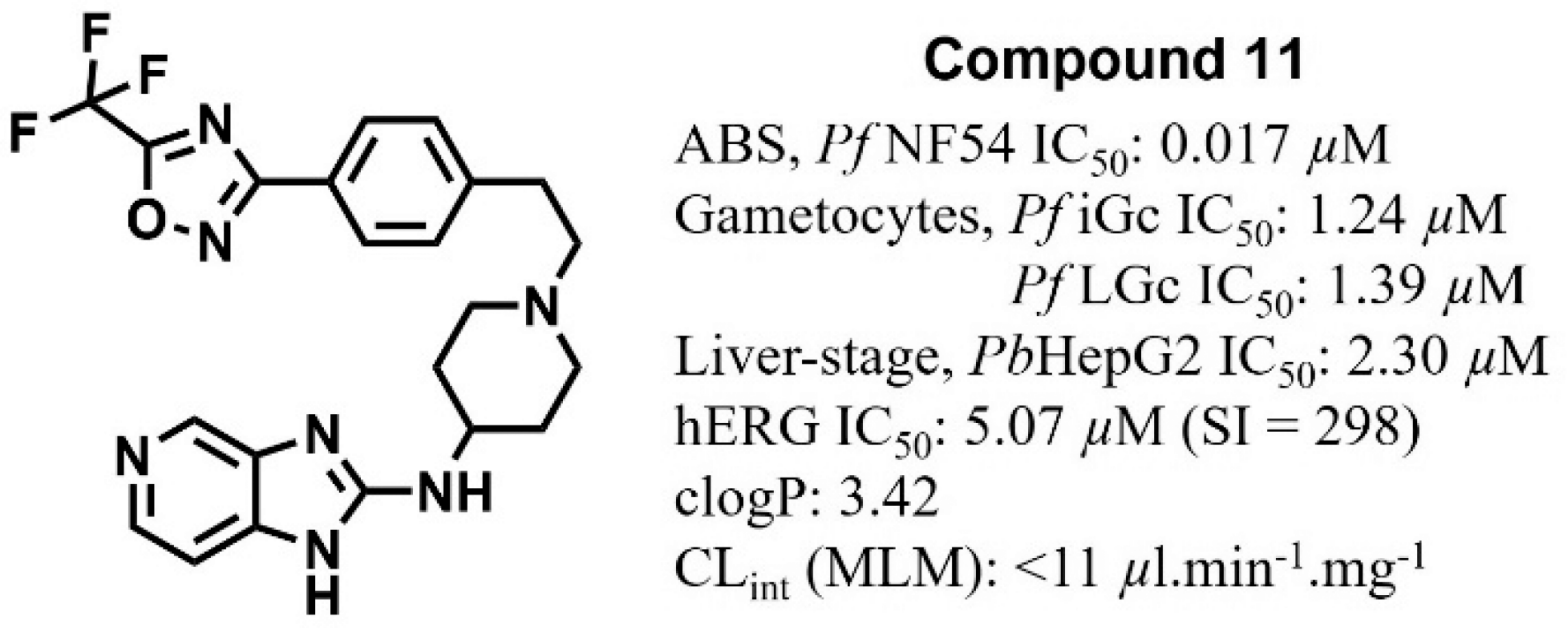

Toward addressing the cardiotoxicity liability associated with the antimalarial drug astemizole (AST, hERG IC_50_ = 0.0042 μM) and its derivatives, we designed and synthesized analogues based on compound **1** (*Pf* NF54 IC_50_ = 0.012 μM; hERG IC_50_ = 0.63 μM), our previously identified 3-trifluoromethyl-1,2,4-oxadiazole AST analogue. Compound **11** retained *in vitro* multistage antiplasmodium activity (ABS *Pf*NF54 IC_50_ = 0.017 μM; gametocytes *Pf*iGc/*Pf*LGc IC_50_ = 1.24/1.39 μM, and liver-stage *Pb*HepG2 IC_50_ = 2.30 μM), good microsomal metabolic stability (MLM CL_int_ < 11 μL·min^–1^·mg^–1^, *E*_H_ < 0.33), and solubility (150 μM). It shows a ∼6-fold and >6000-fold higher selectivity against human ether-á-go-go-related gene higher selectively potential over hERG relative to **1** and AST, respectively. Despite the excellent *in vitro* antiplasmodium activity profile, *in vivo* efficacy in the *Plasmodium berghei* mouse infection model was diminished, attributable to suboptimal oral bioavailability (*F* = 14.9%) at 10 mg·kg^–1^ resulting from poor permeability (log *D*_7.4_ = −0.82). No cross-resistance was observed against 44 common *Pf* mutant lines, suggesting activity via a novel mechanism of action.

The World Health Organization (WHO) reported over 247 million cases of malaria in 2021, with 625 000 related deaths predominantly in children and pregnant women and in Sub-Saharan Africa.^1^ Malaria is a parasitic disease which is primarily caused by *Plasmodium falciparum* (*P. falciparum*, *Pf*) and *Plasmodium vivax* (*P. vivax*, *Pv*) in humans and transmitted via a bite from an infected female *Anopheles* mosquito.^2^ Notwithstanding the effectiveness of the current first-line and standard artemisinin-based combination therapy (ACT) regimens, the rapid emergence of drug resistance is widespread and poses an alarming threat to the current therapeutic options for the treatment of malaria.^3−5^ Efforts in the search for novel, structurally diverse, and affordable drugs have been and must remain an urgent necessity for the control and eradication of malaria.

Drug-induced blockade of the human ether-á-go-go-related gene (hERG) potassium (K^+^) channels is clinically associated with QT prolongation on an electrocardiogram (ECG). Under certain circumstances, this may potentially lead to life-threatening cardiac arrhythmias, i.e., torsades de pointes (TdP).^6,7^ It is for this reason that antihistamine drug astemizole (AST) was withdrawn from the market not long after its discovery and approval (1977). However, its antimalarial properties were later uncovered in a medium throughput screen (MTS) by Chong and colleagues,^21^ resulting in multiple medicinal chemistry efforts by various groups^8−10^ including ours,^11−13^ to reposition AST for malaria by understanding its structure–activity relationships (SAR), structure–property relationships (SPR), improving drug-like properties, and addressing its cardiotoxicity risk using various known strategies.

We recently revealed the identification of compound **1** (Figure 1), a novel structural analogue of AST containing a 3-trifluoromethyl-1,2,4-oxadiazole motif. Compound **1** displayed high *in vitro* antiplasmodium activity (*Pf*NF54/K1 = 0.012/0.040 μM) and demonstrated *in vivo* efficacy in a *Plasmodiumberghei* mouse malaria infection model (*P. berghei*, 99% activity when administered orally at 50 mg·kg^–1^ once daily for 4 days, with mouse survival of 14-days), and a relatively good pharmacokinetic (PK) profile.^13^ Despite its >1000-fold increase in selectivity over hERG K^+^ channels compared to AST, it still possesses a potential cardiotoxicity liability signaled by the high hERG inhibition activity (IC_50_ = 0.63 μM) and a low selectivity index (SI = 53).

**Figure 1:**
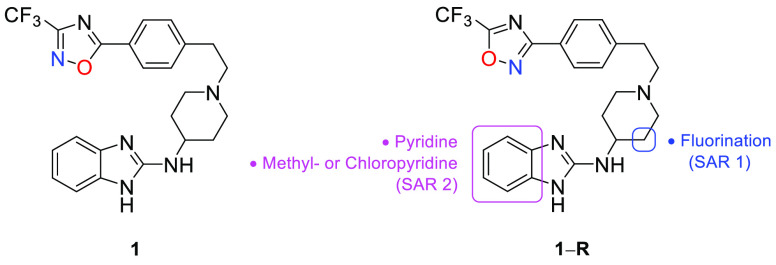
Chemical structure of compound **1** and current SAR exploration.

Henceforth, we sought to use compound **1** as a template to design new analogues with potentially further reduced hERG channel inhibition activity while retaining *in vitro* antiplasmodium potency, antimalarial efficacy, and good absorption distribution metabolism and excretion (ADME) properties. In addition, we wished to evaluate the new analogues with respect to multistage antiplasmodium activity and cross resistance against common *Plasmodium* mutant lines to gain insight into the novelty of the mechanism of action (MoA). Due to easier accessibility to synthetic precursors, we reversed the 3-CF_3_-1,2,4-oxadiazole motif in **1** to its 5-CF_3_-1,2,4-oxadiazole regioisomer **1-R** (Figure 1). This modification was envisaged not to drastically abrogate antiplasmodium activity and physicochemical and metabolism profiles based on previous observations.^13^ Having previously utilized most hERG-affinity reducing strategies,^14,15^ we turned to reducing the basicity of the piperidine 3° nitrogen via β-fluorination (SAR 1, Figure 1) and subtle modifications around the benzimidazole phenyl ring through insertion of a nitrogen atom to generate azabenzimidazoles and concomitantly substituting the benzimidazole ring with previously explored tolerated groups (CH_3_- and Cl-, SAR 2, Figure 1).^13^

The synthesis of target compounds involved *N*,*N*′-dicyclohexylcarbodiimide (DCC)-mediated cyclization of commercially available 1,2-aromatic diamines with appropriately substituted *N*-Boc-protected piperidine isothiocyanates **4a**–**4c** (Scheme 1B,C)^13^ in MeCN to produce 2-amino benzimidazoles **5a**–**5f** in high yields (78–93%).

**Scheme 1:**
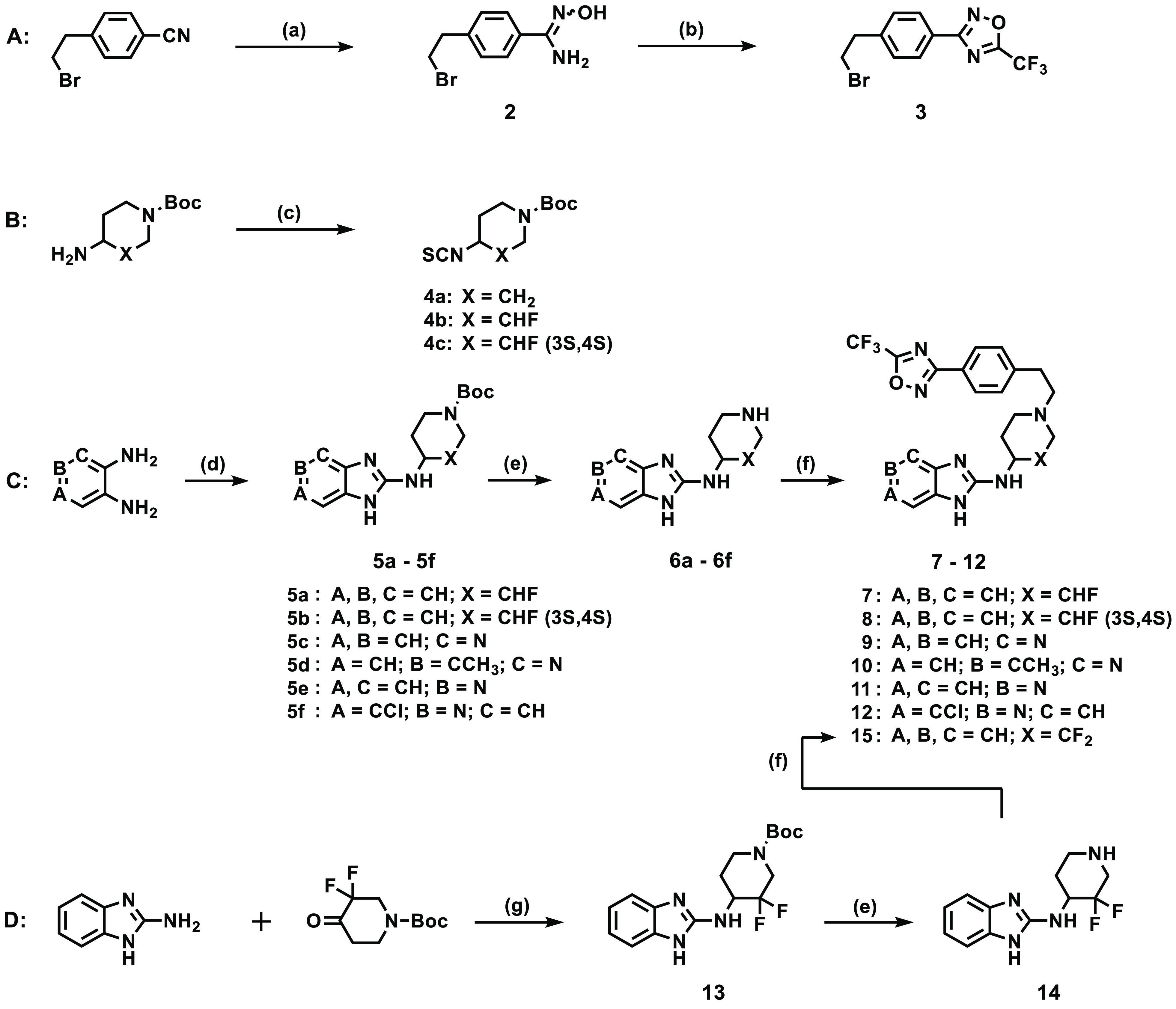
Synthetic Approach for Analogues **7**–**12** and **14** Reagents and conditions: (a) (i) NH_2_OH·HCl, 8-hydroxyquinolone, Et_3_N, ethanol, 79 °C, 1.5 h, (ii) 21 °C, 10% HCl, pH 3 (82%); (b) (CF_3_CO)_2_O, DCM, pyridine, 0–21 °C, 20 min (76%); (c) 1,1-thiocarbonyldiimidazole, DMF, 23 °C, 12 h (42–78%); (d) **4a**–**4c**, DCC, Et_3_N, MeCN, 85 °C, 12 h (78–93%); (e) TFA, DCM, 21 °C, 3 h, then Amberlyst A21 free base, 1 h (95–98%); (f) **3**, MeCN, 85 °C, 8–12 h (43–79%); (g) Ti(O*i*Pr)_4_, Na(OAc)_3_BH, dry THF, 18 °C, 32 h (23%).

*N*-Boc deprotection of **5a**–**5f** using trifluoroacetic acid (TFA), followed by nucleophilic substitution involving the resulting free amines (**6a**–**6f**) and previously prepared **3** (Scheme 1A)^13^ afforded target compounds **7**–**12** (Scheme 1C).^12^ Reductive amination using conventional reagents could not work; we therefore employed the use of titanium(IV) isopropoxide and sodium triacetoxyborohydride (Ti(O*i*Pr)_4_/Na(OAc)_3_BH)^16,17^ in dry THF to deliver *N*-Boc protected intermediate **13** (23%) from 2-aminobenzimidazole and *N*-Boc-3,3-difluoro-4-oxopiperidine (Scheme 1D). Treatment of **13** as previously described (*N-*Boc deprotection then coupling) furnished difluorinated compound **15**.

All target compounds were evaluated for their antiplasmodium activity against the drug sensitive (NF54) strain of *Pf* and for turbidimetric kinetic solubility. Using a discriminatory *Pf*NF54 IC_50_ < 0.20 μM, selected compounds were further screened against the *Pf*K1 multidrug resistant strain. All compounds showing activity *Pf*NF54 IC_50_ < 1.00 μM were tested for their inhibitory activity against the hERG K^+^ channel (Table 1).

**Table 1:**
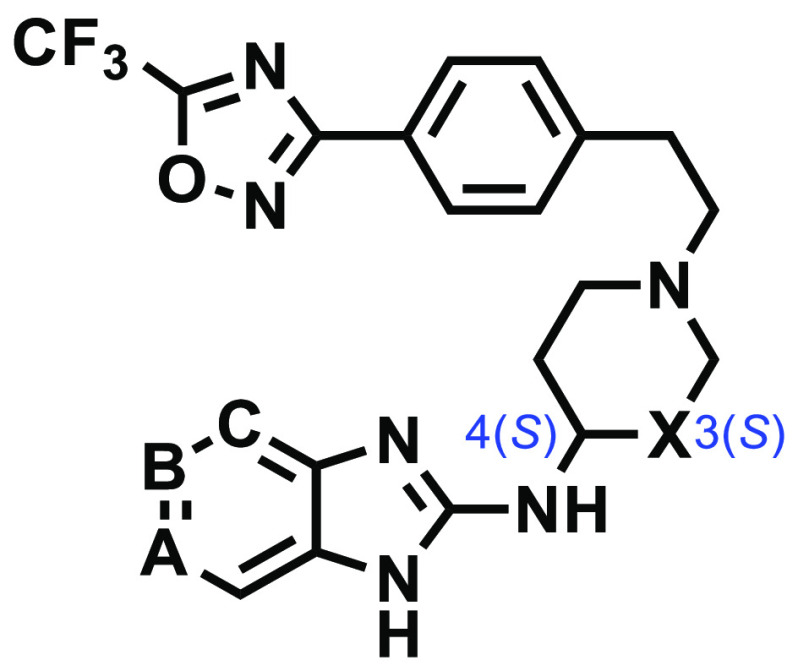
*In Vitro* Antiplasmodium Activity, Solubility, and hERG Channel Inhibition

						*Pf*IC_50_ (μM)^a^				
compd	A	B	C	X	Y	NF54	K1	RI^b^	Sol.^c^ (μM)	hERG^d^ IC_50_, μM (SI^e^)
**7**	CH	CH	CH	CHF	CH_2_	0.219			120	0.98 (4.47)
**8**	CH	CH	CH	CHF(3*S*, 4*S*)	CH_2_	0.147	0.384	2.61	100	
**9**	CH	CH	N	CH_2_	CH_2_	0.244			160	2.72 (11.1)
**10**	CH	CCH_3_	N	CH_2_	CH_2_	0.054	0.082	1.52	120	0.42 (7.78)
**11**	CH	N	CH	CH_2_	CH_2_	0.017	0.033	1.94	160	5.07 (298)
**12**	CCl	N	CH	CH_2_	CH_2_	0.112	0.384	3.42	80	0.83 (7.41)
**15**	CH	CH	CH	CF_2_	CH_2_	0.644			80	1.65 (2.56)
CQ^f^						0.004	0.140	35.0		
verapamil										0.56 ± 0.096

a Mean from *n* ≥ 2 independent experiments with sensitive (NF54) and multidrug-resistant (K1) strains of *P. falciparum*.

b RI: resistance index = [*Pf*K1 IC_50_/*Pf*NF54 IC_50_)].

c Sol.: solubility determined using turbidimetric method in phosphate buffered saline (PBS) at pH 7.4. Hydrocortisone (>200 μM) and reserpine (<10 μM) were used as controls.

d hERG: human ether-a-go-go-related gene.

e SI: selectivity index = [hERG IC_50_/*Pf*NF54 IC_50_]

f CQ: chloroquine.

Reducing the basicity of the piperidine nitrogen via β-fluorination was accompanied by either low (**8**, **15**, *Pf*NF54 IC_50_ = 0.14–0.65 μM) or loss of antiplasmodium activity (**7**, *Pf*NF54 IC_50_ = 2.70 μM). It must, however, be noted that the activity of compound **7** may not represent concise SAR unless separated into respective diastereomers and evaluated. Promisingly, compound **8**, a 3*S*,4*S* diastereomer of **7**, complements the activity of **7**, with a 1.5-fold improvement. β-Fluorinated analogues **7** and **15** demonstrated lower hERG inhibitory activity compared to **1**, albeit no marked improvement in selectivity was observed due to their low antiplasmodium activities (hERG SI = 2.6–6.7).

5-Azabenzimidazole **11** (IC_50_ = 0.017 μM) retained antiplasmodium activity, which was strikingly ∼14 times higher than that of its 4-azabenzimidazole congener **9** (IC_50_ = 0.244 μM). Appealingly, azabenzimidazoles **11** (hERG IC_50_ = 5.07 μM) and **9** (hERG IC_50_ = 2.72 μM) displayed a 4.3- and 8.0-fold decrease in hERG inhibition compared to **1**, respectively, with high aqueous solubility (160 μM). The improved hERG inhibition of **11** (SI = 298) represents a 6200-fold increase in selectivity over hERG compared to that of AST. Interestingly, 5-methyl-4-azabenzimidazole **10** (IC_50_ = 0.054 μM) had a ∼5-fold higher *Pf* activity than unsubstituted **9** (IC_50_ = 0.244 μM) while displaying comparable solubility. Conversely, *Pf* activity and solubility were reduced in 6-chloro-5-azabenzimidazole analogue **12** (IC_50_ = 0.112 μM). In both substituted azabenzimidazole derivatives, α-substitution in the phenyl ring increased hERG inhibitory activity as observed in compounds **10** (5-methyl) and **12** (6-chloro, Table 1).

Cytotoxicity of compounds with *Pf* IC_50_ ≤ 0.1 μM (**10**, **11**, and **12**) was evaluated against the Chinese hamster ovary cell line (CHO) and gametocytocidal activity (Table 2). All three compounds were clean against CHO cells (IC_50_ > 50 μM) indicating an attractive cytotoxicity profile in that cell line.

**Table 2:** *In Vitro* Antiplasmodium Activity against Multiple Life-Cycle Stages and Mammalian Cytotoxicity Profiles

	gametocytes IC_50_, μM				
compd	immature-stage (*Pf*iGc)^a^	late-stage (*Pf*LGc)^a^	liver-stage (*Pb*HepG2 IC_50_, μM)^b^	CHO^c^ IC_50_, μM	CHO SI^d^
**10**	1.62	4.62		>50	>926
**11**	1.24	1.39	2.3 ± 0.4	>50	>2941
**12**	5.85	1.88		>50	>446

a Gametocyte stage: data obtained in a single experiment (*n* = 1) as a technical triplicate. Reference drug: methylene blue (*Pf*iGc IC_50_ = 0.14 μM).

b Liver stage activity: *P. berghei* (*Pb*)-infected HepG2 cell. Data are the mean ± SD of one experiment (*n* = 1), with each concentration tested in triplicate. Reference drug: primaquine (*Pb*HepG2 IC_50_ = 6.0 ± 1.4 μM).

c CHO: Chinese hamster ovary cell line. Reference drug: emetine (CHO IC_50_ = 0.033 μM).

d SI: selectivity index = [CHO IC_50_/*Pf*NF54 IC_50_].

Compound **11** displayed high inhibitory activity against both immature gametocytes (stages I–III, *Pf*iGc IC_50_ = 1.24 μM, Table 2) and late-stage gametocytes (stages IV–V, *Pf*LGc IC_50_ = 1.39 μM), with a comparable immature gametocytes activity with **1** (*Pf*iGc IC_50_ = 1.52 ± 0.3 μM), which nonetheless only displayed specificity against immature Gc’s.^13^ Compounds **10** and **12** equally exhibited high gametocytocidal activities, with specificity (3-fold) toward iGc and LGc, respectively (Table 2). Compound **11** was further profiled for liver-stage activity to augment previous observations in **1** and AST. Liver-stage inhibitory activity (**11**, *Pb*HepG2 IC_50_ = 2.30 μM) was retained, albeit ∼4-fold lower compared to **1** (*Pb*HepG2 IC_50_ = 0.49 μM) and AST (*Pb*HepG2 IC_50_ = 0.59 μM).^13^

Metabolic stability of compounds **9**–**12** showing *Pf*NF54 IC_50_ < 0.10 μM was evaluated using mouse, human and rat liver microsomes (Table 3, data for rat not shown). All tested derivatives were metabolically stable in all microsome species (Table 3). Based on the *in vitro* intrinsic clearance values (CL_int_), low or intermediate *in vivo* hepatic clearance would be expected. Additionally, the microsomal predicted hepatic extraction ratios (*E*_H_ < 0.42, not shown) of each compound were comparable across three species. The experimental partitioning coefficients were generally low to moderate, with log *D*_7.4_ values ranging from −0.1 to 0.25. lipophilic efficiency (LipE) based on log *D*_7.4_ and *in vitro* antiplasmodium activity were generally high and favorable (LipE > 6).^18^

**Table 3:**
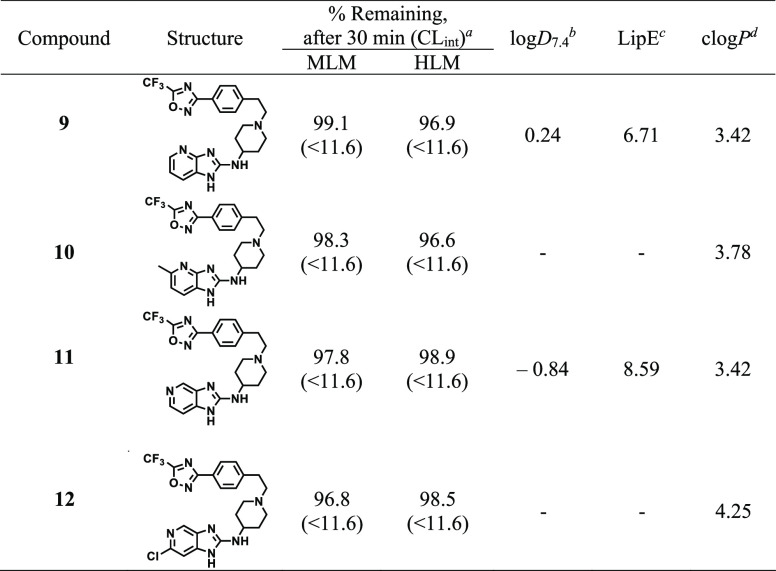
*In Vitro* Microsomal Metabolic Stability of Selected Analogues

a MLM = mouse liver microsomes and HLM = human liver microsomes, expressed as percent (%) of drug remaining after incubation with microsomes for 30 min. CL_int_ = predicted intrinsic clearance in μL·min^–1^·mg^–1^.

b log *D*_7.4_: experimental partitioning coefficient (octanol/water).

c LipE: lipophilic efficiency = pIC_50_(*Pf*NF54) – log *D*_7.4_.

d clogP: calculated lipophilicity, determined using StarDrop software, version 6.5-1. All experimental values are mean values from *n* ≥ 2 independent experiments.

Based on *in vitro* antiplasmodium potency and metabolic stability, compounds **10** and **11** were further evaluated for their *in vivo* efficacy in a *P. berghei* mouse infection model of malaria. However, the standard quadrupole oral dose (po) regimen of 50 mg·kg^–1^ showed moderate percent reduction in parasitemia for **10** (52%) and was suboptimal for **11** (7%), relative to untreated mice (Table 4).

**Table 4:**
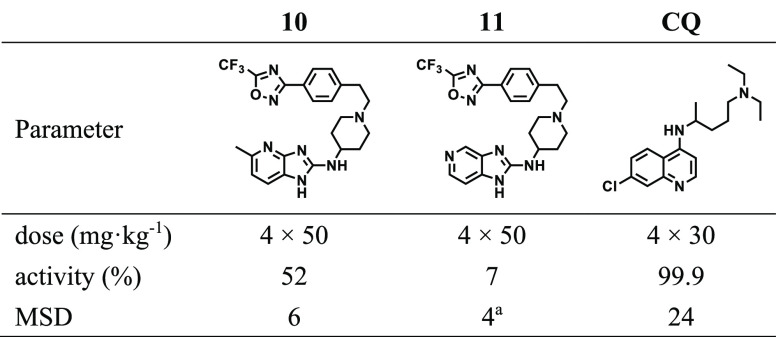
*In Vivo* Efficacy Following Oral Dosing in *P. berghei*-Infected Mice^b^

a Mice were euthanized on day 4 in order to prevent expected death otherwise occurring at day 6 due to high parasitemia.

b MSD = mean survival days; CQ = chloroquine.

When dosed intravenously (iv, 3 mg·kg^–1^), compound **10** displayed rapid clearance from blood (165 mL·min^–1^·kg^–1^, Table 5) with high tissue distribution (43.6 L·kg^–1^) and a relatively short half-life (3.1 h). Orally (10 mg·kg^–1^), compound **10** was rapidly absorbed (*T*_max_ = 0.5 h) with moderately high bioavailability (65.2%) (Table 5). On the other hand, iv dosing of compound **11** (3 mg·kg^–1^) displayed 2.5-fold lower clearance (65.6 mL·min^–1^·kg^–1^, Table 5), with a relatively comparable high tissue distribution (36.1 L·kg^–1^) and a 2-fold higher half-life (6.3 h) than compound **10**.

**Table 5:** Mouse Pharmacokinetic Parameters of **10** and **11**

	**10**		**11**	
parameter	iv	oral	iv	oral
dose (mg·kg^–1^)	3	10	3	10
*C*_max_ (μM)		0.4		0.1
*T*_max_ (h)		0.5		0.7
AUC (μM·min^–1^)	36	85	100	50
*V*_d_ (L·kg^–1^)	43.6		36.1	
CL_int_ (mL·min^–1^·kg^–1^)	165		65.6	
apparent *t*_1/2_ (h)	3.1	3.8	6.3	4.8
*F* (%)		65.2		14.9

The high clearance for these compounds was unexpected given the stability they displayed in microsomes. However, it is notable that these compounds have low log *D* values and high total polar surface areas (TPSA = 96 for both compounds) suggesting that they have poor passive permeability. This chemical space is also associated with transporter-mediated hepatobiliary and renal excretion and this might explain the disconnect between *in vitro* microsomal data and *in vivo* clearance.^19,20^ The free concentration of compound **10** is lower than that of compound **11** (Figure 2) and this likely explains the better *in vivo* efficacy of compound **10.** Notably, when the oral 10 mg/kg data for both compounds is extrapolated to 50 mg/kg, assuming linearity in exposure, the resulting free concentrations are lower than a previously reported AST analogue, which explains the much better *in vivo* efficacy of that compound (99.5% reduction in parasitaemia at the same dose, 4 × 50 mg/kg, as in this work) in the *P. berghei* model.^13^

**Figure 2:**
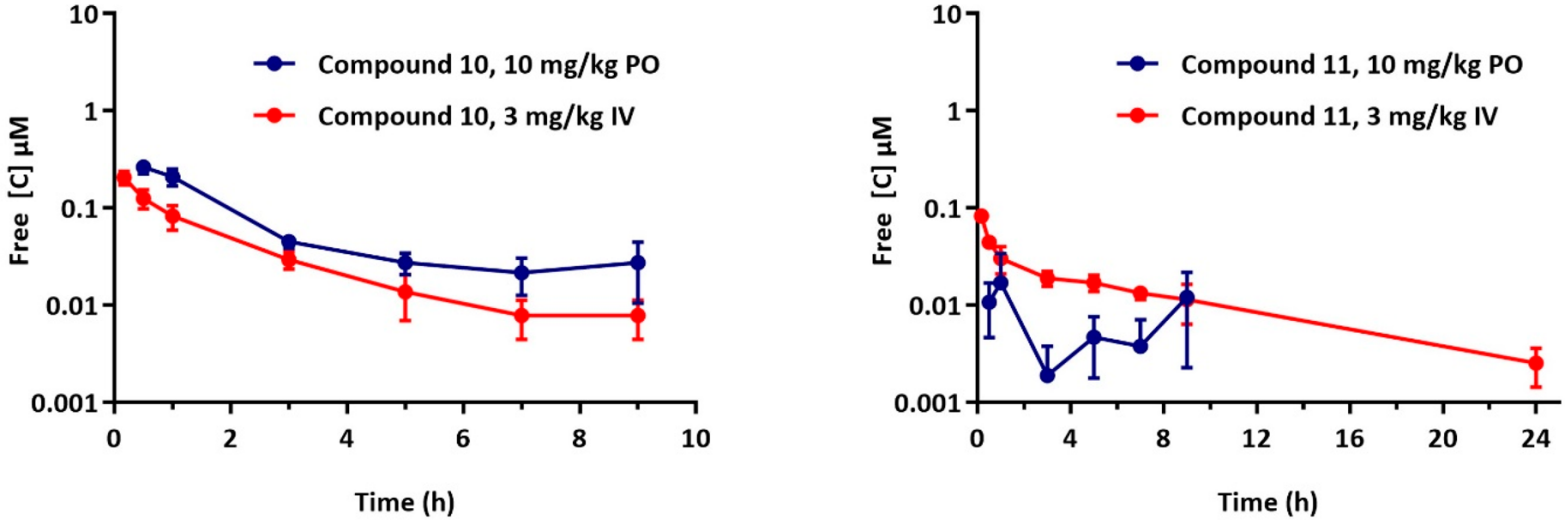
Blood concentrations of compounds **10** and **11** following intravenous (iv) and oral (po) dosing in healthy BalbC mice.

AST and its analogues are known inhibitors of the heme detoxification pathway. The disruption of the machinery in the parasitic hemozoin formation pathway potentially contributes to the antiplasmodium mechanism of action (MoA) of AST and its derivatives.^11−13,21^ To elaborate the current premise, compound **11** was further subjected to an *in vitro* cross-resistance MoA deconvolution study against *P. falciparum* strains covering a range of targets and resistance mechanisms covering 44 uniquely barcoded lines from both the Dd2 and 3D7 genetic backgrounds.^22^ Compound **11** did not show cross-resistance with the mutant lines, suggesting that there is no common resistance phenotype against many of the known mutants, and that **11** potentially acts (*in vitro*) by a novel mechanism or binding mode not represented by the mutations in the pool (Figure S1, Supporting Information).

We have presented SAR exploration of 3-trifluoromethyl-1,2,4-oxadiazole containing AST analogues toward the concomitant reduction of hERG channel inhibitory activity and maintaining antimalarial activity by altering the 1*H*-benzimidazole and 4-aminopiperidine rings. We report the identification of azabenzimidazole analogues that retain antiplasmodium life-cycle activities, good *in vitro* metabolic profile, lower hERG channel inhibition, and a potentially novel mode of action. Although antimalarial activity was diminished, the current frontrunner compounds (**10** and **11**) still represent promising starting points for further optimization. This should be focused on improving the PK profile(s), which would likely influence *in vivo* antimalarial activity. Furthermore, antiplasmodium selectivity over hERG (or *I*_Kr_) channels still require marginal improvement to achieve a clinically acceptable threshold (*C*_max_/hERG IC_50_ > 30).^6^

## Experimental Section

Commercially available chemicals were purchased from Sigma-Aldrich (South Africa and Germany) or Combi-Blocks (United States). ^1^H NMR (all intermediates and final compounds) and ^13^C NMR (for target compounds only) spectra were recorded on Bruker Spectrometer at 300, 400, or 600 megahertz (MHz). Melting points for all target compounds were determined using a Reichert-Jung Thermovar hot-stage microscope coupled to a Reichert-Jung Thermovar digital thermometer (20–350 °C range). Reaction monitoring using analytical thin-layer chromatography (TLC) was performed on aluminum-backed silica-gel 60 F_254_ (70–230 mesh) plates with detection and visualization done using (a) UV lamp (254/366 nm), and (b) iodine vapors. Column chromatography was performed with Merck silica-gel 60 (70–230 mesh). Chemical shifts (δ) are reported in parts per million (ppm) downfield from trimethlysilane (TMS) as the internal standard. Coupling constants (*J*) were recorded in hertz (Hz). Purity of compounds was determined by an Agilent 1260 Infinity binary pump, Agilent 1260 Infinity diode array detector (DAD), Agilent 1290 Infinity column compartment, Agilent 1260 Infinity standard autosampler, and Agilent 6120 quadrupole (single) mass spectrometer, equipped with APCI and ESI multimode ionization source. All compounds tested for biological activity were confirmed to have ≥95% purity by HPLC. No unexpected or unusually high safety hazards were encountered during the experiments.

### General Procedure 1: Synthesis of Intermediates **2** and **3**

The synthetic procedure was followed as previously reported (DOI: 10.1021/acs.jmedchem.2c01516).

### General Procedure 2: Synthesis of Isothiocyanate Intermediates **4a**–**4c**

To a solution of an appropriate *tert*-butyl 4-aminopiperidine-1-carboxylate (1.0 equiv) derivative in DMF at 0 °C was added 1,1′-thiocarbonyldiimidazole (1.10 equiv). The reaction mixture was allowed to rise to room temperature (23 °C) and stirred for 18–20 h at that temperature. The solvent was taken off *in vacuo*, the residue dissolved in EtOAc, and washed with H_2_O (3×). The solvent was removed *in vacuo*, the residue triturated with hexane, and filtered. The filtrate was treated with activated charcoal and filtered through Celite. Removal of solvent afforded pure products.

#### *tert*-Butyl 4-Isothiocyanatopiperidine-1-carboxylate (**4a**)

Obtained from *tert*-butyl 4-aminopiperidine-1-carboxylate (8.00 g, 39.9 mmol) as a colorless oil (7.52 g, 78%). ^1^H NMR (400 MHz, DMSO-*d*_6_) δ 4.11–3.98 (m, 2H), 3.69 (tt, *J* = 11.3, 4.1 Hz, 1H), 3.09–2.94 (m, 2H), 2.17–2.05 (m, 2H), 1.92–1.83 (m, 2H), 1.45 (s, 9H).

#### *tert*-Butyl 3-Fluoro-4-isothiocyanatopiperidine-1-carboxylate (**4b**)

Obtained from *tert*-butyl 4-amino-3-fluoropiperidine-1-carboxylate (1.00 g, 4.60 mmol) as a yellow oil (0.631 g, 53%). ^1^H NMR (400 MHz, DMSO-*d*_6_) δ 4.73 (dtd, *J* = 48.8, 9.1, 4.5, 1H Hz), 4.39 (qd, *J* = 10.3, 4.2 Hz, 1H), 3.61–3.60 (m, 1H), 3.55–3.51 (td, *J* = 12.1, 4.3 Hz, 1H), 3.01–2.90 (m, 2H), 1.83 (dt, *J* = 13.5, 4.2 Hz, 1H), 1.75 (dtd, *J* = 12.1, 9.5, 3.9 Hz, 1H), 1.41 (s, 9H).

#### *tert*-Butyl (3*S*,4*S*)-3-Fluoro-4-isothiocyanatopiperidine-1-carboxylate (**4c**)

Obtained from *tert*-butyl (3*S*,4*S*)-4-amino-3-fluoropiperidine-1-carboxylate (0.650 g, 2.98 mmol) as a yellow oil (0.326 g, 42%). ^1^H NMR (300 MHz, DMSO-*d*_6_) δ 4.68–4.66 (m, 1H), 4.25–4.24 (m, 1H), 3.59–3.57 (m, 1H), 3.53–3.51 (td, *J* = 11.8, 4.2 Hz, 1H), 3.00–2.89 (m, 2H), 1.75 (m, 1H), 1.72 (m, 1H), 1.39 (s, 9H).

## Supporting Information Available

Experimental procedures and characterization data of synthetic intermediates (**5a**–**5f**, **6a**–**6f**, **13**, and **15**) and target compounds (**7**–**12** and **15**); biochemical assay protocols, including solubility and cross-resistance studies; H NMR spectra of representative target compounds (PDF)

## Abbreviations

AST: astemizole
*Pf*iGc: immature *Plasmodium falciparum* gametocytes
*Pf*LGc: mature or late-stage *Plasmodium falciparum* gametocytes
