# Supplementary material for: hERG, Plasmodium Life Cycle, and Cross Resistance Profiling of New Azabenzimidazole Analogues of Astemizole
Source: ACS Med Chem Lett. 2024 Mar 18;15(4):463–9. doi: 10.1021/acsmedchemlett.3c00496 (PMC11017395; doi:10.1021/acsmedchemlett.3c00496)
Supplement: Supplementary file 1 — ml3c00496_si_001.pdf [file ml3c00496_si_001.pdf]

Supplementary Information

hERG, *Plasmodium* Life cycle and Cross Resistance Profiling of New Azabenzimidazole Analogues of Astemizole

Dickson Mambwe,[†] Dina Coertzen,[Ω] Meta Leshabane,[Ω] Mwila Mulubwa,[§] Mathew Njoroge,[§] Liezl Gibhard,[§] Gareth Girling,[♦] Kathryn J. Wicht,[†] Marcus C. S. Lee,[♦,Δ] Sergio Wittlin,[‖,⊥] Diogo Rodrigo Magalhães Moreira,[Ψ] Lyn-Marie Birkholtz,[Ω] and Kelly Chibale[†,‡,§,Π,*]

[†]*Department of Chemistry, University of* Cape *Town, Rondebosch 7701, South Africa.*

[Ω]*Department of Biochemistry, Genetics & Microbiology, Institute for Sustainable Malaria Control, University of Pretoria, Private Bag X20, Hatfield 0028, Pretoria, South Africa.*

[§]*Drug Discovery and Development Centre (H3D), DMPK & Pharmacology, University of Cape Town, Observatory 7925 , South Africa.*

[Ψ]*Centro de Pesquisas Gonçalo Moniz, Fundação Oswaldo Cruz (Fiocruz), Instituto Gonçalo Moniz, CEP 40296-710 Salvador, Brazil.*

[‖]*Swiss Tropical and Public Health Institute, Socinstrasse 57, 4002 Basel, Switzerland.*

[⊥]*University of Basel, 4003 Basel, Switzerland.*

[♦]*Wellcome Sanger Institute, Wellcome Genome Campus, Hinxton, United Kingdom.*

[Δ]*Biological Chemistry and Drug Discovery, School of Life Sciences, University of Dundee, Dundee, Scotland, United Kingdom.*

[Π]*South African Medical Research Council Drug Discovery and Development Research Unit, University of Cape Town, Rondebosch 7701, South Africa.*

[‡]*Institute of Infectious Disease and Molecular Medicine, University of Cape Town, Rondebosch 7701, South Africa.*

Table of Contents

Contents

Compound Synthesis & Characterization 3

General Procedure 3: Synthesis of Intermediates 5a – 5f 3

General Procedure 4: *N*-Boc Deprotection, Synthesis of Intermediates 6a – 6f & 14 5

General Procedure 5: Synthesis of Compounds 7 – 12 & 15 7

Aqueous Solubility 10

In vitro Antiplasmodium Assay 10

In vitro Cytotoxicity Assay 10

In vitro Antigametocytic Activity Assay 11

In vitro Liver-stage Assay (*P. berghei*-sporozoite Infection) 12

In vitro hERG Assay 12

In vivo Antiplasmodium Assay (Swiss TPH) 14

In vivo Pharmacokinetic Studies in Healthy Mice (H3D, DMPK) 14

Barcoded Cross-resistance Studies 15

^{1}H NMR Spectra of Representative Target Compounds 17

References 18

Compound Synthesis & Characterization

Commercially available chemicals were purchased from Sigma-Aldrich (South Africa and Germany) or Combi-Blocks (United States). ^{1}H NMR (all intermediates and final compounds) and ^{13}C NMR (for target compounds only) spectra were recorded on Bruker Spectrometer at 300, 400 or 600 megahertz (MHz). Melting points for all target compounds were determined using a Reichert-Jung Thermovar hot-stage microscope coupled to a Reichert-Jung Thermovar digital thermometer (20 °C – 350 °C range). Reaction monitoring using analytical thin-layer chromatography (TLC) was performed on aluminum-backed silica-gel 60 F_{254} (70−230 mesh) plates with detection and visualization done using (a) UV lamp (254/366 nm), and (b) iodine vapors. Column chromatography was performed with Merck silica-gel 60 (70−230 mesh). Chemical shifts (δ) are reported in parts per million (ppm) downfield from trimethlysilane (TMS) as the internal standard. Coupling constants (J) were recorded in Hertz (Hz). Purity of compounds was determined by an Agilent 1260 Infinity binary pump, Agilent 1260 Infinity diode array detector (DAD), Agilent 1290 Infinity column compartment, Agilent 1260 Infinity standard autosampler, and Agilent 6120 quadrupole (single) mass spectrometer, equipped with APCI and ESI multimode ionization source. All compounds tested for biological activity were confirmed to have ≥95% purity by HPLC. No unexpected or unusually high safety hazards were encountered during the experiments.

General Procedure 3: Synthesis of Intermediates 5a – 5f

To a solution of appropriate 1,2-diamine (1 equiv) in MeCN (15 ml), an appropriate isothiocyanate **4** (1.1 equiv) and Et_3N (1.2 equiv) were added, and the mixture refluxed at 85 °C for 1 hr. DCC (1.2 equiv) was then added, and the reaction mixture was further refluxed at 85 °C for 11 hr. After completion, MeCN was evaporated *in vacuo*, and the residue adsorbed on silica gel. Pure products were obtained after purification *via* flash chromatography using 10% MeOH/DCM as the eluent.

Tert-butyl 4-((1H-benzo[d]imidazol-2-yl)amino)-3-fluoropiperidine-1-carboxylate **(5a).** Obtained from benzene-1,2-diamine (0.200 g, 1.85 mmol) and **4b** (0.530 g, 2.03 mmol), as a white solid (0.482 g, 78%). R_f (6% MeOH/DCM), 0.38. ^{1}H NMR (400 MHz, Methanol-d_4) δ 7.22 (dd, J = 5.9, 3.2 Hz, 2H), 6.98 (dd, J = 5.9, 3.2 Hz, 2H), 4.33 (dtd, J = 48.8, 9.1, 4.2 Hz, 1H), 3.81 (qd, J = 11.2, 4.3 Hz, 1H), 3.33 (d, J = 10.1 Hz, 1H), 2.98 – 2.93 (m, 1H), 2.89 – 2.80 (m, 2H), 2.19 – 2.11 (m, 1H), 1.49

– 1.41 (m, 1H), 1.32 (s, 9H). LC-MS ($APCI^+$/ESI): found *m/z* = 335.2 $[M+H]^+$ (cal. For $C_{17}H_{23}FN_4O_2$, 334.18). Purity: 98%, t_R = 2.365 min.

***Tert-butyl (3S,4S)-4-((1H-benzo[d]imidazol-2-yl)amino)-3-fluoropiperidine-1-carboxylate* (5b).** Obtained from benzene-1,2-diamine (0.100 g, 0.92 mmol) and **4c** (0.265 g, 1.01 mmol), as a white solid (0.255 g, 83%). R_f (6% MeOH/DCM), 0.39. ^1H NMR (400 MHz, Methanol-d_4) δ 7.19 (dd, *J* = 5.8, 3.0 Hz, 2H), 6.93 (dd, *J* = 5.8, 3.0 Hz, 2H), 4.29 – 4.27 (m, 1H), 3.80 – 3.79 (m, 1H), 3.29 – 3.28 (m, 1H), 3.00 – 2.98 (m, 1H), 2.90 – 2.86 (m, 2H), 2.20 – 2.13 (m, 1H), 1.45 – 1.42 (m, 1H), 1.33 (s, 9H). LC-MS ($APCI^+$/ESI): found *m/z* = 335.2 $[M+H]^+$ (cal. For $C_{17}H_{23}FN_4O_2$, 334.18). Purity: 99%, t_R = 2.392 min.

***Tert-butyl 4-((1H-imidazo[4,5-b]pyridine-2-yl)amino)piperidine-1-carboxylate* (5c).** Obtained from pyridine-2,3-diamine (0.200 g, 1.83 mmol) and **4a** (0.489 g, 2.02 mmol), as a an off white solid (0.516 g, 89%). R_f (10% MeOH/DCM), 0.22. ^1H NMR (400 MHz, Methanol-d_4) δ 8.53 (dd, *J* = 5.0, 1.4 Hz, 1H), 7.63 (dd, *J* = 8.2, 1.3 Hz, 1H), 7.19 (dd, *J* = 8.2, 5.0 Hz, 1H), 4.11 – 4.05 (m, 2H), 3.83 (tt, *J* = 10.6, 4.0 Hz, 1H), 3.06 – 2.98 (m, 2H), 2.11 – 2.03 (m, 2H), 1.58 – 1.51 (m, 2H), 1.38 (s, 9H). LC-MS ($APCI^+$/ESI): found *m/z* = 318.2 $[M+H]^+$ (cal. For $C_{16}H_{23}N_5O_2$, 317.19). Purity: 98%, t_R = 2.169 min.

***Tert-butyl 4-((5-methyl-1H-imidazo[4,5-b]pyridin-2-yl)amino)piperidine-1-carboxylate* (5d).** Obtained from 6-methylpyridine-2,3-diamine (0.200 g, 1.62 mmol) and **4a** (0.433 g, 1.78 mmol), as a pale orange solid (0.500 g, 93%). R_f (10% MeOH/DCM), 0.29. ^1H NMR (400 MHz, Methanol-d_4) δ 7.29 (d, J = 8.3 Hz, 1H), 6.50 (d, J = 8.3 Hz, 1H), 4.09 – 4.01 (m, 2H), 3.81 (tt, J = 10.6, 4.0 Hz, 1H), 3.02 – 2.93 (m, 2H), 2.25 (s, 3H), 2.09 – 2.01 (m, 2H), 1.62 – 1.55 (m, 2H), 1.40 (s, 9H). LC-MS ($APCI^+$/ESI): found m/z = 332.2 $[M+H]^+$ (cal. for $C_{17}H_{25}N_5O_2$, 331.20). Purity: 98%, t_R = 2.208 min.

***Tert-butyl 4-((1H-imidazo[4,5-c]pyridin-2-yl)amino)piperidine-1-carboxylate* (5e).** Obtained from pyridine-3,4-diamine (0.200 g, 1.83 mmol) and **4a** (0.489 g, 2.02 mmol), as a white solid (0.481 g, 82%). R_f (10% MeOH/DCM), 0.22. ^1H NMR (400 MHz, Methanol-d_4) δ 8.79 (d, *J* = 1.8 Hz, 1H), 8.23 (dd, *J* = 5.1, 1.8 Hz, 1H), 7.38 (d, *J* = 5.1 Hz, 1H), 4.10 – 4.03 (m, 2H), 3.82 (tt, *J* = 10.8, 4.1 Hz, 1H), 3.09 – 3.01 (m, 2H), 2.14 – 2.08 (m, 2H), 1.60 – 1.53 (m, 2H), 1.43 (s, 9H). LC-MS ($APCI^+$/ESI): found *m/z* = 318.2 $[M+H]^+$ (cal. for $C_{16}H_{23}N_5O_2$, 317.19). Purity: 98%, t_R = 2.098 min.

Tert-butyl 4-((6-chloro-1H-imidazo[4,5-c]pyridin-2-yl)amino)piperidine-1-carboxylate **(5f).** Obtained from 6-chloropyridine-3,4-diamine (0.200 g, 1.39 mmol) and **4a** (0.371 g, 1.53 mmol), as a white solid (0.392 g, 80%). R_f (10% MeOH/DCM), 0.34. ^{1}H NMR (400 MHz, Methanol-d$_4$) δ 8.81 (s, 1H), 7.55 (s, 1H), 4.09 – 4.01 (m, 2H), 3.80 (tt, J = 10.8, 4.1 Hz, 1H), 3.10 – 3.03 (m, 2H), 2.11 – 2.07 (m, 2H), 1.58 – 1.51 (m, 2H), 1.42 (s, 9H). LC-MS (APCI$^+$/ESI): found m/z = 352.2, 354.2 [M+H]$^+$ (cal. for $C_{16}H_{22}ClN_5O_2$, 351.15, 353.14). Purity: 98%, t_R = 2.355 min.

4-((1H-benzo[d]imidazol-2-yl)amino)-3,3-difluoropiperidine-1-carboxylate **(13).** A solution of 2-aminobenzimidazole (0.650 g, 4.88 mmol) and tert-butyl 3,3-difluoro-4-oxopiperidine-1-carboxylate (1.38 g, 5.86 mmol) in anhydrous THF (20 ml) was degassed by purging with N_2 for 15 min. Titanium isopropoxide, Ti(*i*OPr)$_4$ (3.0 equiv) was then added, and the resulting mixture further degassed for 15 min. The reaction was stirred under nitrogen atmosphere for 12 h at 23 °C. When the reaction could not proceed further, sodium triacetoxy borohydride, Na(OAc)$_3$BH (1.0 equiv) was added, and the resulting mixture stirred at 23 °C for 6 hr. The reaction was quenched by adding saturated $NaHCO_3$ (15 ml), after which the resulting mixture was extracted with EtOAc (3 × 30 ml). Combined organic phases were washed with water (2 × 10 ml), and then with brine (10 ml). Pure product was obtained following purification *via* column chromatography with 4 – 5% MeOH/DCM as eluent. White solid (0.395 g, 23%). R_f (6% MeOH/DCM), 0.41. ^{1}H NMR (400 MHz, Methanol-*d*$_4$) δ 7.28 (dd, *J* = 5.6, 3.1 Hz, 2H), 7.01 (dd, *J* = 5.6, 3.1 Hz, 2H), 4.09 – 3.98 (m, 2H), 3.01 – 2.95 (m, 1H), 2.88 – 2.79 (m, 2H), 2.15 – 2.09 (m, 1H), 1.51 – 1.48 (m, 1H), 1.40 (s, 9H). LC-MS (APCI$^+$/ESI): found *m/z* = 353.2 [M+H]$^+$ (cal. For $C_{17}H_{22}F_2N_4O_2$, 352.17). Purity: 98%, t_R = 2.487 min.

General Procedure 4: *N*-Boc Deprotection, Synthesis of Intermediates 6a – 6f & 14

Synthesis of Intermediates 6a – 6d & 14. Respective *N*-Boc protected amines (**5a – 5f** and **13**)were stirred in DCM and TFA (10 equiv) at 23 °C for 2 hr. Following completion, DCM and TFA were evaporated *in vacuo*, and the residue taken up 50% MeOH/DCM. This solution was stirred with Amberlyst® A-21 free base resin at room temperature (23°C) until pH was neutral. The mixture was then filtered, and the filtrate evaporated *in vacuo* to afford free amines.

N-(3-fluoropiperidin-4-yl)-1H-benzo[d]imidazol-2-amine **(6a).** Obtained from **5a** (0.470 g, 1.41 mmol) as a white solid (0.319 g, 97%). R_f (10% MeOH/DCM), 0.19. ^{1}H NMR (400 MHz, Methanol-*d*$_4$) δ 7.19 (dd, *J* = 5.5, 3.1 Hz, 2H), 6.85 (dd, *J* = 5.5, 3.1 Hz, 2H), 4.30 (dtd, *J* = 49.3, 10.3, 4.5 Hz,

1H), 3.79 (qd, J = 11.0, 4.2 Hz, 1H), 3.18 (d, J = 11.6 Hz, 1H), 2.90 – 2.81 (m, 1H), 2.73 – 2.58 (m, 2H), 2.03 – 1.91 (m, 1H), 1.44 – 1.36 (m, 1H). LC-MS ($APCI^+$/ESI): found m/z = 235.1 $[M+H]^+$ (cal. For $C_{12}H_{15}FN_4$, 234.13). Purity: 98%, t_R = 0.295 min.

N-((3S,4S)-3-fluoropiperidin-4-yl)-1H-benzo[d]imidazol-2-amine **(6b).** Obtained from **5b** (0.210 g, 0.63 mmol) as a white solid (0.143 g, 97%). R_f (10% MeOH/DCM), 0.20. ^1H NMR (400 MHz, Methanol-d_4) δ 7.21 (dd, J = 5.8, 3.3 Hz, 2H), 6.88 (dd, J = 5.8, 3.3 Hz, 2H), 4.28 – 4.27 (m, 1H), 3.81 – 3.79 (m, 1H), 3.19 (d, J = 10.9 Hz, 1H), 2.95 – 2.87 (m, 1H), 2.75 – 2.61 (m, 2H), 2.09 – 1.95 (m, 1H), 1.45 – 1.38 (m, 1H). LC-MS ($APCI^+$/ESI): found m/z = 235.1 $[M+H]^+$ (cal. For $C_{12}H_{15}FN_4$, 234.13). Purity: 99%, t_R = 0.301 min.

N-(piperidin-4-yl)-1H-imidazo[4,5-b]pyridin-2-amine **(6c).** Obtained from **5c** (0.480 g, 1.51 mmol) as a white solid (0.322 g, 98%). R_f (10% MeOH/DCM), 0.18. ^1H NMR (400 MHz, Methanol-d_4) δ 8.39 (dd, J = 5.3, 1.5 Hz, 1H), 7.58 (dd, J = 8.0, 1.5 Hz, 1H), 7.13 (dd, J = 8.0, 5.3 Hz, 1H), 3.89 (tt, J = 10.9, 3.8 Hz, 1H), 3.95 – 3.86 (m, 2H), 3.08 – 2.98 (m, 2H), 2.03 – 1.94 (m, 2H), 1.54 – 1.43 (m, 2H). LC-MS ($APCI^+$/ESI): found m/z = 218.1 $[M+H]^+$ (cal. for $C_{11}H_{15}N_5$, 217.13). Purity: 98%, t_R = 0.359 min.

5-methyl-N-(piperidin-4-yl)-1H-imidazo[4,5-b]pyridin-2-amine **(6d).** Obtained from **5d** (0.470 g, 1.42 mmol) as a pale orange solid (0.321 g, 98%). R_f (10% MeOH/DCM), 0.18. ^1H NMR (400 MHz, Methanol-d_4) δ 7.21 (d, J = 8.1 Hz, 1H), 6.53 (d, J = 8.1 Hz, 1H), 3.90 (tt, J = 10.8, 3.9 Hz, 1H), 3.98 – 3.89 (m, 2H), 3.01 – 2.93 (m, 2H), 2.33 (s, 3H), 2.05 – 1.96 (m, 2H), 1.59 – 1.50 (m, 2H). LC-MS ($APCI^+$/ESI): found m/z = 232.2 $[M+H]^+$ (cal. for $C_{12}H_{17}N_5$, 231.15). Purity: 98%, t_R = 0.298 min.

N-(piperidin-4-yl)-1H-imidazo[4,5-c]yridine-2-amine **(6e).** Obtained from **5e** (0.450 g, 1.42 mmol) as a white solid (0.300 g, 98%). R_f (10% MeOH/DCM), 0.12. ^1H NMR (400 MHz, Methanol-d_4) δ 8.56 (d, J = 1.5 Hz, 1H), 8.19 (dd, J = 5.3, 1.5 Hz, 1H), 7.29 (d, J = 5.3 Hz, 1H), 3.91 (tt, J = 10.9, 4.0 Hz, 1H), 4.01 – 3.92 (m, 2H), 2.98 – 2.89 (m, 2H), 2.08 – 1.97 (m, 2H), 1.59 – 1.51 (m, 2H). LC-MS ($APCI^+$/ESI): found m/z = 218.1 $[M+H]^+$ (cal. For $C_{11}H_{15}N_5$, 217.13). Purity: 98%, t_R = 0.246 min.

6-chloro-N-(piperidin-4-yl)-1H-imidazo[4,5-c]yridine-2-amine (6f). Obtained from **5f** (0.370 g, 1.05 mmol) as a white solid (0.252 g, 95%). R_f (10% MeOH/DCM), 0.19. ^1H NMR (400 MHz,

Methanol-d_4) δ 8.56 (s, 1H), 7.66 (s, 1H), 3.89 (tt, *J* = 10.7, 3.9 Hz, 1H), 3.96 – 3.85 (m, 2H), 3.05 – 2.93 (m, 2H), 2.08 – 1.99 (m, 2H), 1.56 – 1.48 (m, 2H). LC-MS ($APCI^+$/ESI): found *m/z* = 252.1, 254.1 $[M+H]^+$ (cal. For $C_{11}H_{14}ClN_5$, 251.09, 253.09). Purity: 99%, t_R = 0.256 min.

***N-(3,3-difluoropiperidin-4-yl)-1H-benzo[d]imidazol-2-amine* (14).** Obtained from **13** (0.350 g, 1.00 mmol) as a white solid (0.245 g, 98%). R_f (10% MeOH/DCM), 0.21. ^1H NMR (400 MHz, Methanol-d_4) δ 7.21 (dd, *J* = 5.6, 3.2 Hz, 2H), 6.98 (dd, *J* = 5.6, 3.2 Hz, 2H), 4.11 – 3.99 (m, 2H), 2.99 – 2.86 (m, 1H), 2.65 – 2.49 (m, 2H), 2.11 – 2.05 (m, 1H), 1.48 – 1.39 (m, 1H). LC-MS ($APCI^+$/ESI): found *m/z* = 253.1 $[M+H]^+$ (cal. for $C_{12}H_{14}F_2N_4$, 252.12). Purity: 98%, t_R = 0.356 min.

General Procedure 5: Synthesis of Compounds 7 – 12 & 15

A solution of appropriate amine (**6a – 6f** & **14**, 1.0 equiv) and K_2CO_3 (1.5 equiv) in MeCN was stirred under reflux at 80 °C for 30 minutes. To this solution, **3** (1.2 equiv) was added, and the mixture further stirred under reflux at 85 °C for 5 – 24 hours. After completion, MeCN was taken off under reduced pressure, the residue taken up in 10% MeOH/DCM and filtered. The filtrate was adsorbed on silica gel, after which column chromatography was performed using a 3 – 10% MeOH/DCM gradient as eluent, to afford final compounds.

***N-(3-fluoro-1-(4-(3-(trifluoromethyl)-1,2,4-oxadiazol-5-yl)phenethyl)piperidin-4-yl)-1H-benzo[d]imidazol-2-amine* (7).** Obtained from **6a** (0.200 g, 0.85 mmol) and **3** (0.329 g, 1.03 mmol) as a pale yellow solid (0.286 g, 71%). m.p.: 84 – 86 °C; R_f (10% MeOH/DCM) 0.45. ^1H NMR (600 MHz, Methanol-d_4) δ 8.03 (d, *J* = 7.8 Hz, 2H), 7.44 (d, *J* = 7.8 Hz, 2H), 7.21 (dd, *J* = 6.2, 3.1 Hz, 2H), 6.99 (dd, *J* = 6.2, 3.1 Hz, 2H), 4.49 (dtd, *J* = 49.6, 9.1, 4.5 Hz, 1H), 3.83 (qd, *J* = 10.3, 4.7 Hz, 1H), 3.30 – 3.22 (m, 1H), 3.00 – 2.85 (m, 3H), 2.79 – 2.69 (m, 2H), 2.39 – 2.26 (m, 2H), 2.19 (dt, *J* = 14.3, 4.4 Hz, 1H), 1.61 (dtd, *J* = 11.6, 9.2, 3.9 Hz, 1H). ^{13}C NMR (101 MHz, Methanol-d_4) δ ^{13}C NMR (101 MHz, Methanol-d_4) δ 169.10, 154.51, 145.13, 136.93, 129.39 (2C), 127.37 (2C), 122.80 (2C), 120.25 (2C), 111.38 (q, *J* = 28.5 Hz), 91.08, 89.31, 58.73, 55.70 (d, *J* = 19.5 Hz), 54.28 (d, *J* = 36.3), 51.05, 32.82, 29.57. LC-MS ($APCI^+$/ESI): found m/z = 475.2 $[M+H]^+$ (cal. for $C_{23}H_{22}F_4N_6O$, 474.18). Purity: 99%, t_R = 2.597 min.

***N-((3S,4S)-3-fluoro-1-(4-(5-(trifluoromethyl)-1,2,4-oxadiazol-3-yl)phenethyl)piperidin-4-yl)-1H-benzo[d]imidazol-2-amine* (8).** Obtained from **6b** (0.100 g, 0.43 mmol) and **3** (0.165 g, 0.51 mmol) as a pale yellow solid (0.132 g, 65%). m.p.: 79 – 80 °C; R_f (10% MeOH/DCM) 0.49. ^1H NMR (600

MHz, Methanol-d_4) δ 7.98 (d, *J* = 8.0 Hz, 2H), 7.49 (d, *J* = 8.0 Hz, 2H), 7.19 (dd, *J* = 5.9, 3.2 Hz, 2H), 7.01 (dd, *J* = 5.9, 3.2 Hz, 2H), 4.52 – 4.51 (m, 1H), 3.84 – 3.82 (m, 1H), 3.33 – 3.27 (m, 1H), 3.08 – 2.84 (m, 3H), 2.81 – 2.72 (m, 2H), 2.41 – 2.29 (m, 2H), 2.18 – 2.10 (m, 1H), 1.58 (dtd, *J* = 12.1, 8.9, 3.8 Hz, 1H). ^{13}C NMR (101 MHz, Methanol-d_4) δ ^{13}C NMR (101 MHz, Methanol-d_4) δ 171.12, 155.56, 145.33, 136.78, 129.09 (2C), 127.88 (2C), 122.12 (2C), 120.29 (2C), 112.07 (q, *J* = 29.1 Hz), 92.00, 89.08, 58.55, 55.34 (d, *J* = 19.1 Hz), 54.20 (d, *J* = 33.0), 51.01, 32.94, 29.41. LC-MS (APCI$^+$/ESI): found m/z = 475.2 $[M+H]^+$ (cal. for $C_{23}H_{22}F_4N_6O$, 474.18). Purity: 98%, t_R = 2.611 min.

N-(1-(4-(3-(trifluoromethyl)-1,2,4-oxadiazol-5-yl)phenethyl)piperidin-4-yl)-1H-imidazo[4,5-b]pyridin-2-amine **(9).** Obtained from **6c** (0.050 g, 0.23 mmol) and **3** (0.089 g, 0.28 mmol) as a pale yellow solid (0.079 g, 75%). m.p.: 88 – 90 °C; R_f (10% MeOH/DCM), 0.22. ^{1}H NMR (400 MHz, Methanol-d_4) δ 8.33 (dd, *J* = 5.4, 1.4 Hz, 1H), 7.90 (d, *J* = 8.1 Hz, 2H), 7.45 – 7.32 (m, 3H), 7.19 (dd, *J* = 7.8, 5.4 Hz, 1H), 3.96 (tt, *J* = 10.2, 4.0 Hz, 1H), 3.12 – 3.01 (m, 2H), 2.95 – 2.86 (m, 2H), 2.73 – 2.66 (m, 2H), 2.38 – 2.27 (m, 2H), 2.10 – 2.01 (m, 2H), 1.69 – 1.58 (m, 2H). ^{13}C NMR (101 MHz, Methanol-d_4) δ 171.15, 158.65, 152.24, 148.89, 140.53, 138.65, 130.25 (2C), 128.33 (2C), 124.58, 122.30, 118.69, 116.58 (q, *J* = 32.9 Hz), 60.32, 51.62, 49.58 (2C), 34.55, 31.57 (2C). LC-MS (APCI$^+$/ESI): found *m/z* = 458.2 $[M+H]^+$ (cal. for $C_{22}H_{22}F_3N_7O$, 457.18). Purity: 98%, t_R = 2.106 min.

5-methyl-N-(1-(4-(3-(trifluoromethyl)-1,2,4-oxadiazol-5-yl)phenethyl)piperidin-4-yl)-1H-imidazo[4,5-b]pyridin-2-amine **(10).** Obtained from **6d** (0.100 g, 0.43 mmol) and **3** (0.166 g, 0.52 mmol) as a pale-yellow solid (0.158 g, 78%). m.p.: 105 – 107 °C; R_f (10% MeOH/DCM), 0.32. ^{1}H NMR (600 MHz, Methanol-d_4) δ 8.05 (d, *J* = 8.3 Hz, 2H), 7.47 (d, *J* = 8.3 Hz, 2H), 7.38 (d, *J* = 7.9 Hz, 1H), 6.83 (d, *J* = 7.9 Hz, 1H), 3.79 (tt, *J* = 10.6, 4.3 Hz, 1H), 3.19 – 3.09 (m, 2H), 3.02 – 2.92 (m, 2H), 2.86 – 2.78 (m, 2H), 2.54 – 2.41 (m, 5H), 2.18 – 2.09 (m, 2H), 1.75 – 1.66 (m, 2H). ^{13}C NMR (101 MHz, Methanol-d_4) δ 172.23, 165.23, 155.32, 145.30, 136.95, 130.01 (2C), 128.32 (2C), 121.88, 119.52, 118.52 (q, *J* = 29.3 Hz), 111.02, 61.32, 51.25 (2C), 50.20, 43.65, 34.08, 31.29 (2C), 24.19. LC-MS (APCI$^+$/ESI): found *m/z* = 472.2 $[M+H]^+$ (cal. For $C_{23}H_{24}F_3N_7O$, 471.20). Purity: 98%, t_R = 2.324 min.

N-(1-(4-(3-(trifluoromethyl)-1,2,4-oxadiazol-5-yl)phenethyl)piperidin-4-yl)-1H-imidazo[4,5-c]pyridin-2-amine **(11).** Obtained from **6e** (0.100 g, 0.46 mmol) and **3** (0.178 g, 0.55 mmol) as a

pale-yellow solid (0.158 g, 75%). m.p.: 145 – 147 °C; R_f (10% MeOH/DCM), 0.23. ^{1}H NMR (600 MHz, Methanol-d_4) δ 8.49 (d, *J* = 0.8 Hz, 1H), 8.23 (d, *J* = 6.5 Hz, 1H), 8.11 (d, *J* = 8.0 Hz, 2H), 7.60 (d, *J* = 6.5 Hz, 1H), 7.56 (d, *J* = 8.0 Hz, 2H), 4.23 – 4.15 (m, 1H), 3.79 – 3.70 (m, 2H), 3.52 – 3.42 (m, 2H), 3.28 – 3.20 (m, 2H), 2.45 – 2.35 (m, 2H), 2.16 – 2.02 (m, 2H). ^{13}C NMR (101 MHz, Methanol-d_4) δ 179.32, 166.53, 148.5, 147.50, 140.58, 138.96, 137.56, 133.56, 129.39 (2C), 127.45 (2C), 123.56, 116.08 (q, *J* = 28.5 Hz), 111.32, 62.98, 52.89, 49.32 (2C), 33.58, 32.08 (2C). LC-MS (APCI$^+$/ESI): found *m/z* = 458.2 [M+H] $^+$ (cal. for $C_{22}H_{22}F_3N_7O$, 457.18). Purity: 98%, t_R = 2.196 min.

***6-chloro-N-(1-(4-(3-(trifluoromethyl)-1,2,4-oxadiazol-5-yl)phenethyl)piperidin-4-yl)-1H-imidazo[4,5-c]pyridin-2-amine* (12).** Obtained from **6f** (0.100 g, 0.40 mmol) and **3** (0.152 g, 0.47 mmol) as a pale-yellow solid (0.155 g, 79%). m.p.: 113 – 114 °C; R_f (10% MeOH/DCM), 0.33. ^{1}H NMR (600 MHz, Methanol-d_4) δ 8.51 (s, 1H), 8.06 (d, *J* = 7.9 Hz, 2H), 7.48 (d, *J* = 7.9 Hz, 2H), 7.29 (s, 1H), 4.02 (tt, *J* = 11.0, 4.2 Hz, 1H), 3.55 (dt, *J* = 12.8, 3.9 Hz, 2H), 3.33 – 3.27 (m, 2H), 3.20 – 3.13 (m, 2H), 3.05 (td, *J* = 12.5, 4.0 Hz, 2H), 2.31 – 2.23 (m, 2H), 1.92 (dtd, *J* = 14.0, 11.9, 4.1 Hz, 2H). ^{13}C NMR (101 MHz, Methanol-d_4) δ 177.23, 167.23, 162.35, 147.56, 139.56, 138.85, 137.33, 132.41, 129.09 (2C), 128.02 (2C), 122.30, 115.95 (q, *J* = 29.6 Hz), 110.25, 63.25, 51.62, 50.25 (2C), 34.65, 31.24 (2C). LC-MS (APCI$^+$/ESI): found *m/z* = 492.1 [M+H] $^+$ (cal. For $C_{22}H_{21}ClF_3N_7O$, 491.14). Purity: 98%, t_R = 2.454 min.

N-(3,3-difluoro-1-(4-(3-(trifluoromethyl)-1,2,4-oxadiazol-5-yl) phenethyl) piperidin-4-yl)-1H-benzo[d]imidazol-2-amine (15). Obtained from **14** (0.050 g, 0.20 mmol) and **3** (0.076 g, 0.24 mmol) as a yellow solid (0.042 g, 43%). m.p.: 87 – 89 °C; R_f (10% MeOH/DCM) 0.46. ^{1}H NMR (400 MHz, Methanol-d_4) δ 7.95 (d, *J* = 8.1 Hz, 2H), 7.52 (d, *J* = 8.1 Hz, 2H), 7.26 (dd, *J* = 6.0, 2.9 Hz, 2H), 6.92 (dd, *J* = 6.0, 2.9 Hz, 2H), 4.08 (m, 1H), 3.35 – 3.23 (m, 1H), 3.05 – 2.85 (m, 3H), 2.80 – 2.69 (m, 2H), 2.37 – 2.24 (m, 2H), 2.19 (td, *J* = 13.5, 3.9 Hz, 1H), 1.61 (dtd, *J* = 12.0, 9.3, 4.0 Hz, 1H). ^{13}C NMR (101 MHz, Methanol-d_4) δ ^{13}C NMR (101 MHz, Methanol-d_4) δ 171.23, 155.23, 146.32, 135.62, 129.85 (2C), 128.05 (2C), 123.56, 123.01, 121.53 (2C), 119.65 (2C), 113.53 (q, *J* = 29.0 Hz), 96.32, 88.56, 59.08, 56.32 (t, *J* = 23.2 Hz), 53.21 (t, *J* = 28.3), 50.32, 33.32, 28.95. LC-MS (APCI$^+$/ESI): found m/z = 493.2 [M+H]$^+$ (cal. For $C_{23}H_{21}F_5N_6O$, 492.17). Purity: 99%, t_R = 2.499 min.

Aqueous Solubility. Solubility was measured from amorphous solid forms of the compounds using the turbidimetric method. Following the dissolution of test compound in DMSO to make a 10 mM stock solution, a pre-dilution plate was prepared by taking from each stock solution and serially diluting in triplicate to yield concentrations from 0.25 mM to 10.0 mM on a 96 well plate. From each pre-dilution solution, secondary dilutions of the compounds in both DMSO and 0.01M pH 7.4 PBS were prepared in triplicate on a second 96-well plate. Wells in columns 1-6 would contain compounds in DMSO, while those in columns 7 – 12 would contain samples in PBS at similar nominal concentrations as those in DMSO. The final volume of solvent in each assay plate was 200 µl, prepared by pipetting 4 µl each of solution from the pre-dilution plate to the corresponding well into both DMSO and PBS (both 196 µL). This ensures that the final concentration of DMSO in the PBS aqueous buffer does not exceed 2% v/v. Similarly, a second secondary plate containing compound concentrations of 60, 100 and 120 µM was also prepared. Different concentrations in DMSO were prepared as controls to determine false turbidimetric absorbance readings arising from the compounds in solution absorbing incident radiation at the test wavelength. Following preparation, the assay plate was covered and left to equilibrate for 2 h at 25 °C. Afterwards, UV-vis absorbance readings from the plate were measured at 620 nm using a SpectraMax 340PC^{384} microplate reader. Plots of corrected absorbance against compound concentration were computed for a graphical representation of the data using MS Excel. Reserpine and hydrocortisone were used as positive and negative controls, respectively.

***In vitro* Antiplasmodium Assay**. The compounds were tested using the modified [^{3}H]-hypoxanthine incorporation assay, as previously reported.[1]

***In vitro* Cytotoxicity Assay**. Compounds were screened against Chinese Hamster Ovarian (CHO) mammalian cell lines, using the 3-(4,5-dimethylthiazol-2-yl)-2,5-diphenyltetrazoliumbromide (MTT) assay.[2] Emetine was used as the reference standard. It was prepared to 2 mg/ml in distilled water, while the stock solutions of test compounds were prepared to 20 mg/ml in DMSO (100%), with the highest concentration of solvent to which the cells were exposed having no measurable effect on the cell viability. The initial concentration of the compounds and control was 100 µg/ml, which was serially diluted in complete medium with 10-fold dilutions to give 6 concentrations, the lowest being 0.001 µg/ml. Plates were incubated for 48 h with 100 µl of test compound and 100 µl of cell suspension in each well and developed afterward by adding 25 µl of sterile MTT (Thermo

Fisher Scientific) to each well, followed by 4 h of incubation in the dark. The plates were then centrifuged, the medium aspirated, and 100 µl of DMSO was added to dissolve crystals before reading the absorbance at 540 nm. Data were analyzed, and the sigmoidal dose−response was derived using GraphPad Prism version 4.0 software. All experiments were performed as three independent biological repeats, each with technical triplicates.

***In vitro* Antigametocytic Activity Assay**. Gametocytes were produced as per the method reported by Reader and co-workers.[3] The luciferase reporter assay was used to enable accurate, reliable and quantifiable investigations of the stage-specific action of gametocytocidal compounds for the early and late gametocyte marker cell line NF54-*Pf*S16-GFP-Luc. Assays were set up on day 5 and 10 (representing >90% of either early stage II/III or mature/late stage IV/V gametocytes, respectively). In each instance, assays were set up using a 2 – 3% gametocytemia, 1.5% hematocrit culture and 48 h drug pressure in a gas chamber (90% N_2, 5% O_2, and 5% CO_2) at 37°C. Luciferase activity was determined in 30 µl parasite lysates by adding 30 µl luciferin substrate (Promega Luciferase Assay System) at room temperature and detection of resultant bioluminescence at an integration constant of 10s with the GloMax® Explorer Detection System with Instinct® Software. Methylene blue (5 µM) and internal project specific controls were included as controls. The dual point screens were performed as technical triplicates for a single biological assay.

Table S1: *In vitro* gametocytocidal activity of selected analogues (duo-point and IC_{50}'s).

Compound	Early Gametocytes (EG)^a			Late Gametocytes (LG)^b		
	% activity		*Pf*EG IC_{50} (µM)	% activity		*Pf*LG IC_{50} (µM)
	1.0 µM	5.0 µM		1.0 µM	5.0 µM	
**AST**	5	61	-	0<br>13^c	11<br>52^c	3.35^c
**7**	0	27.6 ± 0.8	8.16	10.9 ± 2.3	37.8 ± 0.5	8.00
**9**	0.5	1.60 ± 2.7	8.32	6.0 ± 10.4	0	>20
**10**	39.1	66.5 ± 4.7	1.62	1.1 ± 1.8	56.2 ± 7.7	4.62
**11**	40.6 ± 0.2	85.8 ± 1.4	1.24	30.9 ± 5.0	83.0 ± 1.7	1.39
**12**	3.4 ± 2.6	23.4 ± 4.6	5.85	31.1 ± 3.5	66.5 ± 2.0	1.88

^{a}*Pf*NF54 early-stages (I – III) and ^{b}late-stages (IV – V) gametocytes, obtained at 1.0 µM and 5.0 µM (n = 3, technical triplicates). Methylene blue (EG luc at 1.0 µM = 95% inhibition, EG IC_{50} = 0.2 µM; LG luc at 1.0 µM = 92% inhibition, LG IC_{50} = 0.14 µM).^{c}Data generated using ATP bioluminescence assay. ^{d}n = 3, technical triplicates ± SEM. ND = not determined.

***In vitro* Liver-stage Assay (*P. berghei*-sporozoite Infection)**. In a 24-well plate, HepG2 was seeded onto monolayers to a 5 × 10^3 cell number in 250 µl of RPMI medium. After 24 h of incubation, medium was replaced by a volume 250 µl of 5 × 10^4 *P. berghei*-GFP sporozoites per well. After 1 h, medium was replaced by 250 µl of medium containing drugs and further incubated for 48 h at 37 °C. Primaquine biphosphate (Sigma-Aldrich) and Astemizole (USP reference standard) were employed as reference drugs. Negative control wells received medium only. 48 h post-infection, cells were trypsinized and the percentage (%) of infected cells was determined by flow cytometry (BD LSRFortessa) using at least 10000 events. For determining host cell viability, in a 96-well plate, HepG2 was seeded onto monolayers to a 5 × 10^3 cell number in 100 µl of RPMI medium. After 24 h of incubation, a volume 100 µl of medium containing drugs was added and further incubated at 37 °C. Negative control wells received medium only and positive wells received gentian violet. After 48 h of drug exposure, cell viability was measured using CellTiter Glo (Promega). Two independent experiments were performed. Drugs were tested in five different concentrations, each one in triplicate.

***In vitro* Metabolic Stability Assay**. This assay was performed in duplicate using a 96-well microtiter plate. Test compounds (0.1 µM) were incubated at 37 °C in mouse and pooled human liver microsomes with a final protein concentration of 0.4 $mg.ml^{-1}$; XenoTech, Lenexa, KS suspended in 0.1 M phosphate buffer at pH 7.4 for predetermined time points. This was in the presence and absence of cofactor-reduced nicotinamide adenine dinucleotide phosphate (NADPH, 1.0 mM). The reactions were quenched by adding ice-cold MeCN containing an internal standard (Carbamazepine, 0.0236 µg/ml). The samples were centrifuged, and the supernatant was analyzed via liquid chromatography-tandem mass spectrometry (LC–MS/MS) (Agilent Rapid Resolution HPLC, AB SCIEX 4500 MS). The relative loss of the parent compound with time was monitored, and plots were prepared for each compound of Ln% remaining versus time to determine the first-order rate constant for compound depletion. This was used to calculate the degradation half-life and subsequently to predict the *in vitro* intrinsic clearance (CL_{int}) and *in vitro* hepatic extraction ratio (E_H).[4]

***In vitro* hERG Assay**. A QPatch hERG assay employing a four-point concentration response format was used to carry out hERG inhibition studies by Metrion Biosciences Ltd, Cambridge, UK. The hERG gene was stably expressed in a CHO K1 cell line from the American Type Culture Collection

(ATCC) which was grown and passaged under standard culture conditions. The external (e) and internal (i) recording solutions were of the following compositions (mM): NaCl – 140(e) : 0 (i); KCl – 2 (e): 70 (i); KF – 0 (e) : 60 (i); HEPES – 10 (e) : 10 (i); $MgCl_2$ – 1 (e) : 0 (i); $CaCl_2$ –2 (e) : 0 (i); Glucose – 5 (e) : 0 (i); EGTA - 0 (e) : 5 (i); Mg_2ATP – 0 (e) : 5 (i) and pH – 7.4 (NaOH) (e) : 7.2 (KOH) (i). The external recording solution was regularly prepared and kept at 4°C until required and was maintained at room temperature during recording. The internal recording solution was prepared and kept at –20 °C until required.

The QPatch is a chip-based planar patch clamp which is automated. Using suction, cells added to each well are drawn across a small aperture creating a Giga-ohm seal between the membrane surface and a treated silicon surface. A small volume of bathing solution containing the test compound or control bathing solution is added to a reservoir on the chip which perfuses across the cell through quartz-lined microfluidic channels. The solution is removed by capillary action before the next sample is added. Using the industry +40/–40 voltage protocol, currents were triggered from a holding potential of –90 mV at a stimulus frequency of 0.1 Hz.

By cumulatively adding four escalating concentrations of the test compounds to an individual cell, the concentration response curves were established. This was done by firstly allowing the whole-cell configuration to be achieved followed by the addition of the vehicle (0.1% DMSO v/v in external recording solution) to each well in two bolus additions allowing a two-minute recording time between each addition. This was followed by the addition of four concentrations (0.3 – 10 μM) of test compounds in two bolus additions at 2-minute intervals. The effect on the hERG tail current amplitude was measured during the 4-minute recording time. The concentrations (0.3, 1, 3 and 10 μM) of the test samples were prepared in such a way to have a final concentration of 0.1% of DMSO v/v in the external recording solution. For each compound, the experiments at each concentration were done in triplicate and using a bioinformatics suite developed and running in Pipeline Pilot (Biovia, USA), the percent inhibition, as a reduction in mean peak current relative to the value measured at the end of the vehicle control period, was calculated. Such percent inhibition data were used to construct the concentration-response curves which enabled calculation of the IC_{50} values. For compounds which could not achieve 50% inhibition even at the highest tested concentration of 10 μM, extrapolated IC_{50} values for such are reported. In this regard, all IC_{50} values above 10 μM reported in this article were extrapolated and should be treated with caution.

***In vivo* Antiplasmodium Assay (Swiss TPH)**. In vivo efficacy was assessed as previously described,[5] with the modification that mice (n = 3) were infected with a GFP-transfected P. berghei ANKA strain (donated by A. P. Waters and C. J. Janse, Leiden University, The Netherlands), and parasitemia was determined using standard flow cytometry techniques. The detection limit was 1 parasite in 1000 erythrocytes (that is, 0.1%). The activity was calculated as the difference between the mean percent parasitemia for the control and treated groups expressed as a percent relative to the control group. Compounds were dissolved or suspended in a vehicle consisting of 70% Tween-80 and 30% ethanol, followed by a 10-fold dilution in H_2O and oral administration as four consecutive daily doses (4, 24, 48, and 72 h after infection). Blood samples for the quadruple-dose regimens were collected on day 4 (96 h after infection). The survival time in days was also recorded up to 30 days after infection. A compound was considered curative if the animal survived to day 30 after infection with no detectable parasites by slide reading.

In vivo studies conducted at the Swiss TPH, Basel were approved by the veterinary authorities of the Canton Basel-Stadt (Permit No. 1731 and 2303) based on Swiss Cantonal (Verordnung Veterinäramt Basel-Stadt) and National Regulations (The Swiss Animal Protection Law, Tierschutzgesetz).

***In vivo* Pharmacokinetic Studies in Healthy Mice (H3D, DMPK).** Male Balb/c mice were used for animal pharmacokinetics evaluations. All animal work was carried out in accordance with the University of Cape Town's ethics policies using protocols approved by the research ethics committee (AEC022_00). Compounds were administered intravenously as a bolus of DMA/PEG/PPG (10/30/60) and orally in 0.5% (w/v) HPMC with 0.2% Tween-80 aqueous solution to male Balb/c mice (n = 3 for the oral group, and n = 3 for the intravenous group). Mice were not fasted overnight and were allowed to eat *ad libitum*. Animals were permitted access *ad libitum* to water.

PK Sampling: Blood samples were collected from the tail vein of the mice into heparinized microcentrifugation tubes at predetermined sampling times (0.17, 0.5, 1, 3, 5, 7, 9, and 24 h for intravenous dosing; 0.5, 1, 3, 5, 7, 9, and 24 h for oral dosing). All samples were stored at −80 °C until extraction.

Bioanalytical Method: Frozen whole blood was thawed at room temperature. Ten microliters of samples were extracted by protein precipitation using 100 µL of acetonitrile containing a 10 ng/mL internal standard (MMV394902) and centrifuged. Calibration

standards and quality controls were extracted following the same procedure. Supernatants were injected onto the column for LC–MS/MS analysis. The analytical limit of quantitation (LOQ) was 2 ng/mL. The accuracy, precision, and recovery for each study were within acceptable limits.

Instrument:	AB SCIEX 5500 QTRAP equipped with a Turbo V™ ion source coupled to an Agilent 1260 HPLC.
Detection:	Positive electrospray ionization under MRM scans
Column:	Kinetex C18, 50 × 2.1 mm, 2.6 μM
LC Conditions:	Gradient 0.4 ml/min, 7 min run, injection volume 5 μl, column temperature 40℃, sample tray temperature 8℃
Mobile Phase:	A: 0.1% formic acid; B 0.1% formic acid in acetonitrile
Analysis Software:	Analyst® 1.6.3 software for instrument control, data acquisition and analysis.

Calculation of Pharmacokinetics Parameters: PK parameters were calculated by noncompartmental analysis using PK Solutions 2.0 (Summit Research Services, Montrose, CO, USA) with a method based on curve stripping.

Barcoded Cross-resistance Studies. Resistance was assessed as previously described.[6] The parasite pool of resistant mutants consisted of 44 barcoded lines, generated via CRISPR/Cas9 genome-editing to integrate unique barcodes into a nonessential gene (pfpare/PF3D7_0709700), covering both the chloroquine-resistant Dd2 and chloroquine-sensitive 3D7 genetic backgrounds, including wild-type (WT) lines for both strains. The pool of freshly thawed mutant lines was exposed to compound **11** at 3 × IC_{50} for 14 days alongside an untreated control pool of the same lines grown without drug pressure. The proportions of mutant lines remaining in the treated vs untreated pools were measured via next generation sequencing to track the unique barcodes and quantified as the $\log_2$ of the fold change in proportions. The competitive outgrowth of any line with log2 fold change > 2.5 signified cross resistance, while those measuring < 2.5 showed a significant sensitivity to the compound. In the case of **11**, no lines were found to be resistant to the compound, while four lines showed slight sensitivities ($\log_2$ fold change -2.6 to -3.0), including lines with mutations in the P-type cation translocating ATPase PfATP4 and the cytochrome bc1 complex.

Figure S1: The log$_2$fold change of the untreated vs compound **11**-treated barcoded mutant pools. LCF = log$_2$ fold change; 3D7g = the chloroquine-sensitive 3D7 strain; Dd2 = the chloroquine-resistant Dd2 strain; WT= wild-type.

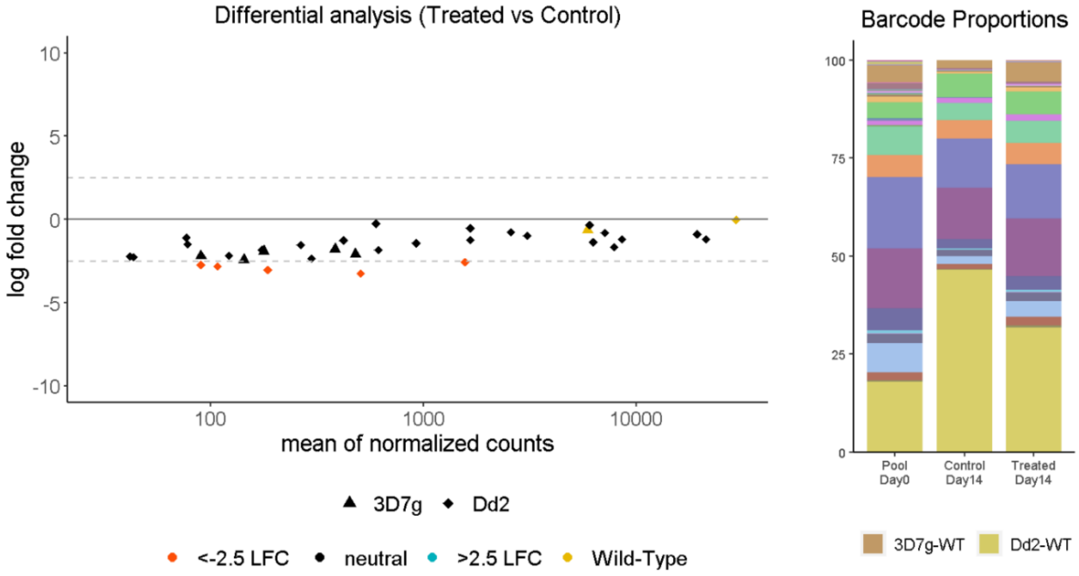

^{1}H NMR Spectra of Representative Target Compounds

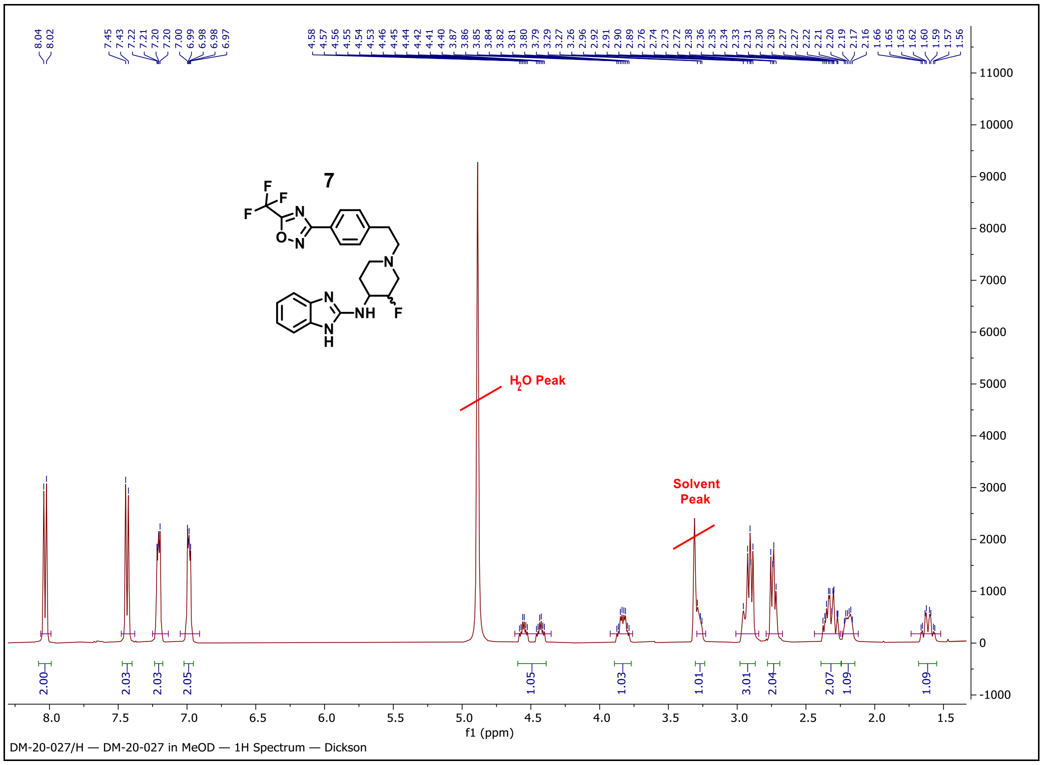

Figure S2: ^{1}H-NMR Spectrum of Compound **7** in Methanol-d_4 at 400 MHz

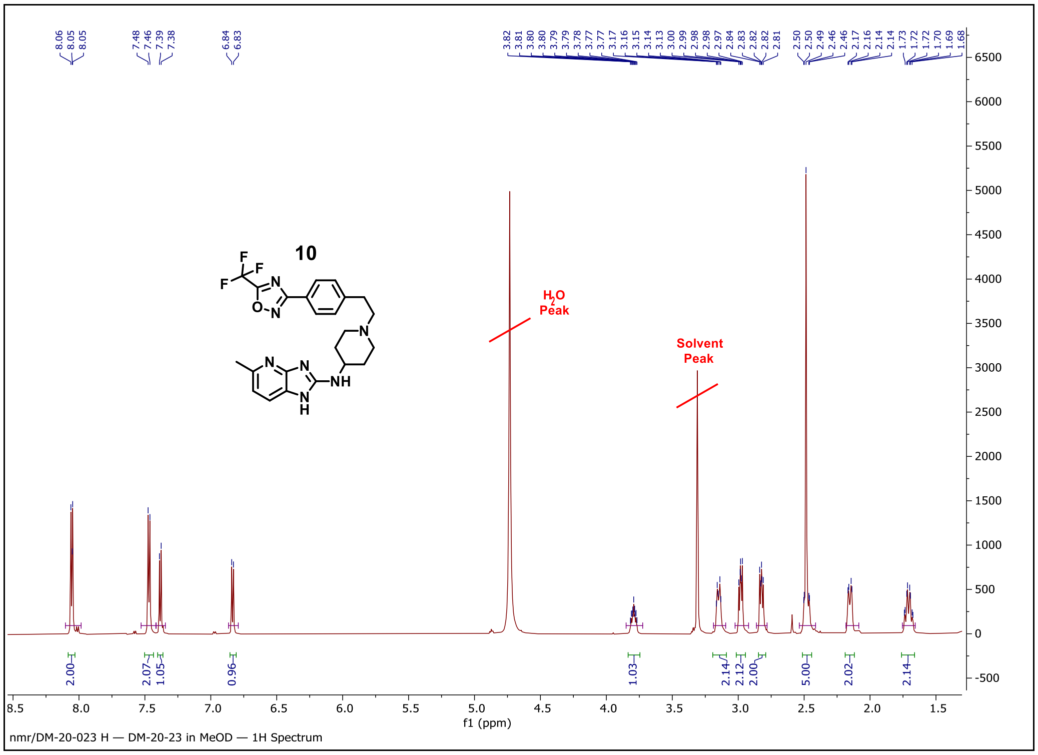

Figure S3: ^{1}H-NMR Spectrum of Compound **10** in Methanol-d_4 at 600 MHz

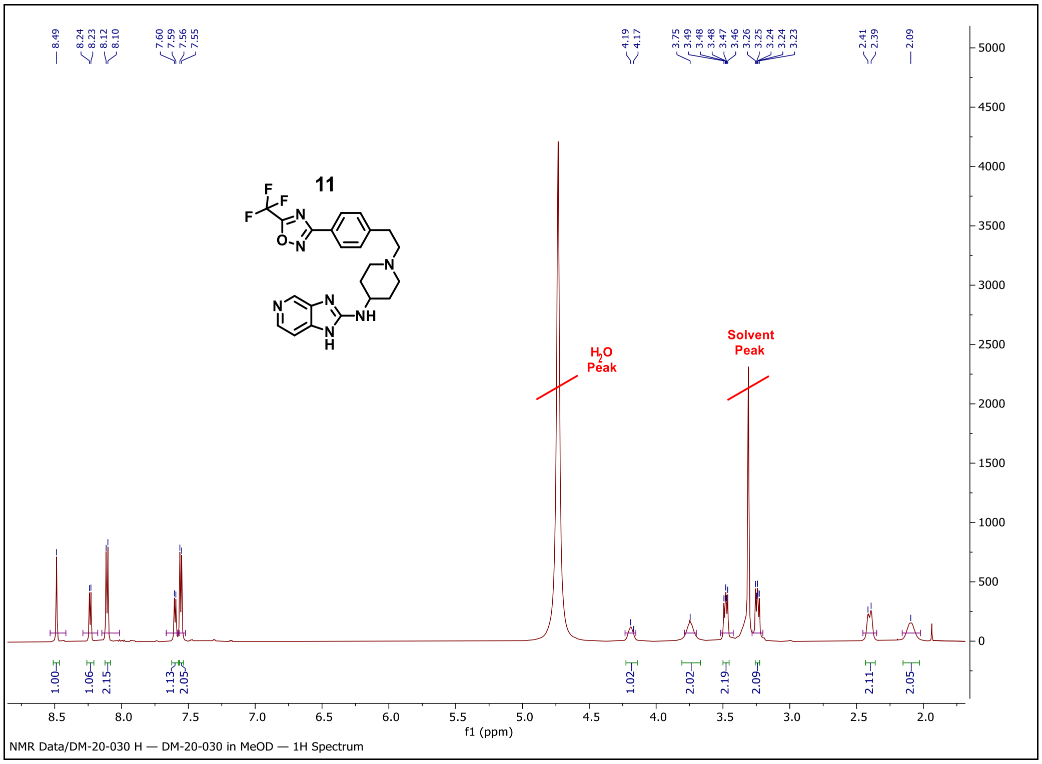

Figure S4: ^{1}H-NMR Spectrum of Compound **11** in Methanol-d_4 at 600 MHz

References

(1) Snyder, C.; Chollet, J.; Santo-Tomas, J.; Scheurer, C.; Wittlin, S. In Vitro and in Vivo Interaction of Synthetic Peroxide RBx11160 (OZ277) with Piperaquine in Plasmodium Models. *Exp. Parasitol.* **2007**, *115* (3), 296–300.

(2) Liu, Y.; Peterson, D. A.; Kimura, H.; Schubert, D. Mechanism of Cellular 3-(4,5-Dimethylthiazol-2-Yl)-2,5-Diphenyltetrazolium Bromide (MTT) Reduction. *J. Neurochem.* **2002**, *69* (2), 581–593.

(3) Reader, J.; Botha, M.; Theron, A.; Lauterbach, S. B.; Rossouw, C.; Engelbrecht, D.; Wepener, M.; Smit, A.; Leroy, D.; Mancama, D.; et al. Nowhere to Hide: Interrogating Different Metabolic Parameters of Plasmodium Falciparum Gametocytes in a Transmission Blocking Drug Discovery Pipeline towards Malaria Elimination. *Malar. J.* **2015**, *14* (1), 213.

(4) Bertrand, M.; Jackson, P.; Walther, B. Rapid Assessment of Drug Metabolism in the Drug Discovery Process. *Eur. J. Pharm. Sci.* **2000**, *11*, S61–S72.

(5) González Cabrera, D.; Douelle, F.; Younis, Y.; Feng, T.-S.; Le Manach, C.; Nchinda, A. T.;

Street, L. J.; Scheurer, C.; Kamber, J.; White, K. L.; et al. Structure–Activity Relationship Studies of Orally Active Antimalarial 3,5-Substituted 2-Aminopyridines. *J. Med. Chem.* **2012**, *55* (24), 11022–11030.

(6) Carrasquilla, M.; Drammeh, N. F.; Rawat, M.; Sanderson, T.; Zenonos, Z.; Rayner, J. C.; Lee, M. C. S. Barcoding Genetically Distinct Plasmodium Falciparum Strains for Comparative Assessment of Fitness and Antimalarial Drug Resistance. *MBio* **2022**, *13* (5).
